# Targeting Cancer Stem Cells and Their Niche: Current Therapeutic Implications and Challenges in Pancreatic Cancer

**DOI:** 10.1155/2017/6012810

**Published:** 2017-08-06

**Authors:** Jiangang Zhao, Jiahui Li, Hans A. Schlößer, Felix Popp, Marie Christine Popp, Hakan Alakus, Karl-Walter Jauch, Christiane J. Bruns, Yue Zhao

**Affiliations:** ^1^Department of General, Visceral and Cancer Surgery, University of Cologne, Cologne, Germany; ^2^Department of General, Visceral und Vascular Surgery, Ludwig-Maximilian-University (LMU), Munich, Germany; ^3^Cologne Interventional Immunology, University of Cologne, Cologne, Germany; ^4^Department of General, Visceral und Vascular Surgery, Otto von Guericke University, Magdeburg, Germany

## Abstract

Cancer stem cells (CSCs) have been identified as a subpopulation of stem-like cancer cells with the ability of self-renewal and differentiation in hematological malignancies and solid tumors. Pancreatic cancer is one of the most lethal cancers worldwide. CSCs are thought to be responsible for cancer initiation, progression, metastasis, chemoresistance, and recurrence in pancreatic cancer. In this review, we summarize the characteristics of pancreatic CSCs and discuss the mechanisms involved in resistance to chemotherapy, the interactions with the niche, and the potential role in cancer immunoediting. We propose that immunotherapy targeting pancreatic CSCs, in combination with targeting the niche components, may provide a novel treatment strategy to eradicate pancreatic CSCs and hence improve outcomes in pancreatic cancer.

## 1. Introduction

Pancreatic ductal adenocarcinoma, referred to in this review as pancreatic cancer, is one of the most lethal malignancies around the world. In 2012, an estimated 338,000 new cases were diagnosed and 330,000 deaths occurred worldwide [1]. Despite advances in the diagnosis and treatment of pancreatic cancer, there has been little improvement in the survival of the patients over the past two decades [2, 3]. The 5-year survival for all stages of pancreatic cancer diagnosed from 2007 to 2013 is 8.2% in the USA [4]. Pancreatic cancer continues to be a challenging disease. Radical resection remains the only potentially curative treatment. However, more than 50% of patients are diagnosed locally advanced or metastatic and only 15–20% of patients have resectable disease at the time of diagnosis [5]. Nevertheless, a significant proportion of patients who undergo surgical resection followed by adjuvant therapy will experience recurrence [6]. To date, chemotherapy is the main treatment option for patients with advanced pancreatic cancer [7, 8]. Several clinical trials have shown a modest survival benefit, such as FOLFIRINOX (oxaliplatin, irinotecan, leucovorin, and fluorouracil) and nab-paclitaxel plus gemcitabine [9, 10]. Improved understanding of the interactions between pancreatic cancer cells and the tumor microenvironment (TME) provides valuable therapeutic targets for pancreatic cancer [11]. For instance, targeting tumor-associated macrophages (TAMs) with CCR2 inhibition in combination with FOLFIRINOX in patients with borderline resectable and locally advanced pancreatic cancer has shown encouraging results with moderate toxicity in a phase Ib trial [12]. However, the clinical efficacy of systemic chemotherapy and molecular-targeted therapies, such as EGFR and VEGFR inhibition, in the management of pancreatic cancer is still considered unsatisfactory [13–15]. Therefore, exploring mechanisms involved in pancreatic cancer evolution is urgently required. Increasing evidence supports the idea that a subpopulation of pancreatic cancer cells, called pancreatic cancer stem cells (CSCs), plays a significant role in the process of tumor initiation, local invasion, distant metastasis, chemoresistance, and relapse in pancreatic cancer [16, 17]. Therapeutic approaches to target CSCs are expected to have widespread clinical implications for pancreatic cancer treatment.

## 2. Overview of Pancreatic CSCs

The existence of CSCs and their role remained obscure largely due to technological challenges for a long time [18, 19]. During the past two decades, numerous studies have provided support for this concept. In 1997, Bonnet and Dick first identified CD34^++^CD38^−^ cells as CSCs in human acute myeloid leukemia [20]. Since then, CSCs have been identified in various solid tumors including breast cancer, brain tumor, pancreatic cancer, melanoma, head and neck cancer, and colorectal cancer [21–27]. All these findings reveal that CSCs, a subpopulation of cancer cells with the ability to self-renew and the capacity to proliferate and differentiate, are the driving force for cancer initiation, progression, metastasis, and chemoresistance [28–30].

Pancreatic CSCs were first identified in 2007. Li et al. established human pancreatic cancer xenografts in NOD/SCID mice. After 16 weeks, xenografts were digested and sorted for the markers of CD44, CD24, and epithelial-specific antigen (ESA)/epithelial cell adhesion molecule (EpCAM). Sorted cells were then injected into NOD/SCID mice. They identified a subpopulation of pancreatic cancer cells with the specific cell surface markers CD44^+^CD24^+^ESA^+^ as pancreatic CSCs, which showed stem-cell-like properties of self-renewal, the ability to produce differentiated progeny, and upregulation of developmental signaling molecule sonic hedgehog [24]. Then, Hermann et al. demonstrated CD133 as a cell surface marker of pancreatic CSCs. CD133^+^ pancreatic cancer cells were highly tumorigenic and resistant to gemcitabine. As few as 500 CD133^+^ pancreatic cancer cells were capable of forming orthotopic tumors in athymic mice, but 10^6^ CD133^−^ cells did not result in any tumor formation. Elimination of CD133^+^CXCR4^+^ pancreatic cancer cells significantly reduced the metastatic potential of pancreatic cancer [31]. In 2010, Rasheed et al. identified aldehyde dehydrogenase (ALDH) expression as a marker for pancreatic CSCs. ADLH-positive pancreatic cancer cells showed enhanced clonogenic growth and high migratory ability, which had a negative impact on the overall survival of patients with pancreatic cancer [32]. In 2011, Li et al. identified c-Met as a new marker for pancreatic CSCs. c-Met^high^ pancreatic cancer cells could form spheres and c-Met inhibitor or knockdown of c-Met significantly inhibited tumor sphere formation in vitro. c-Met^high^ cells had increased tumorigenic potential in mice. They established human pancreatic cancer xenografts in NOD/SCID mice and found that administration of c-Met inhibitors could inhibit tumor growth, reduce the population of pancreatic CSCs, and prevent metastases when given alone or in combination with gemcitabine [33]. In 2014, Bailey et al. described microtubule regulator, doublecortin and Ca^2+^/calmodulin-dependent kinase-like 1 (DCLK1) as a morphologically and functionally distinct population of pancreatic CSCs. Pancreatic cancer cells expressing DCLK1 displayed high clonogenic potential. Inhibition of *γ*-secretase activity reduced the abundance of these cells in murine pancreatic intraepithelial neoplasia (PanIN) and prevented PanIN progression [34]. Fujiwara et al. identified CD166 expression as another important characteristic of tumorigenicity and invasive and migratory activities of pancreatic cancer cells. CD166^+^ pancreatic cancer cells were more tumorigenic, while CD166^−^ cells exhibited stronger invasive and migratory activities [35].

In addition to the identification of specific phenotypes, several studies aim to characterize of pancreatic CSCs based on gene expression analysis. Bao et al. reported that pancreatic CSCs (CD44^+^/CD133^+^/EpCAM^+^) exhibited differential expression of more than 1600 mRNAs, including *BMP4*, *FoxQ1*, Sox4, and *Wnt3a*, compared with CD44^−^/CD133^−^/EpCAM^−^ cells. The knockdown of FoxQ1 in pancreatic CSCs resulted in the inhibition of aggressive behaviour [36]. Skoda et al. identified 602 differentially expressed genes in pancreatic CSCs (CD24^+^/CD44^+^/EpCAM^+^/CD133^+^), including upregulated Wnt signaling (WNT2, WNT2B, FZD6, and FZD7), upregulation of LYN expression, and downregulation of FYN expression [37]. These differentially expressed genes are supposed to be essential for regulating functions and phenotypes of pancreatic CSCs. Recently, a study using a combined approach with high-sensitivity mutation detection and whole-transcriptome analysis of the same single cell to characterize CSCs in patients with chronic myeloid leukemia during treatment with tyrosine kinase inhibitors provides insights into disease evolution and points to new therapeutic targets [38]. This method which exemplifies how single-cell analysis can identify CSCs might be applied to other cancers, including pancreatic cancer.

**Figure 1:**
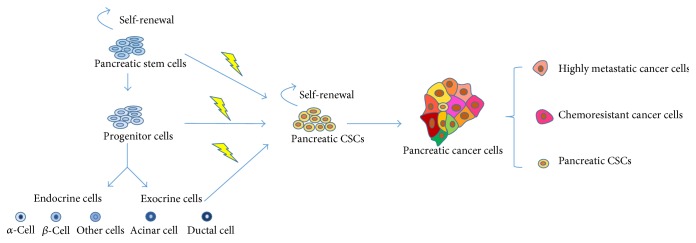
The origin of pancreatic CSC hypothesis. Normal stem cells give rise to progenitor cells that proliferate and differentiate into various types of mature cells, including *α*-cells, *β*-cells, acinar cells, and ductal cells. Pancreatic CSCs may originate from the transformation of normal stem cells or progenitor cells through the accumulation of mutations. On the other hand, under certain conditions, pancreatic ductal cells and acinar cells may acquire genetic alterations and dedifferentiate into pancreatic CSCs. Pancreatic CSCs have the ability of self-renewal and differentiation. Finally, pancreatic CSCs and their differentiated progeny contribute to tumor heterogeneity.

According to the two most common models, intratumoral heterogeneity arises hierarchically and stochastically. These models explain CSCs from different perspectives and are not mutually exclusive [39]. Here, we mainly discuss the hierarchical model (Figure 1()). According to this model, carcinogenesis occurs when stem cells, progenitor cells, or differentiated cells give rise to CSCs. Even though much effort has been made to identify and characterize pancreatic CSCs, the origin of pancreatic CSCs is still widely unknown [40]. One hypothesis is that pancreatic CSCs may originate from stem cells or progenitor cells that reside in normal tissues with accumulating mutations, which ultimately trigger a malignant transformation [41]. Pancreatic islets are formed by self-duplication of adult cells, and their formation does not rely on stem cells [42]. However, this does not preclude the existence of stem cells in the pancreas. On the other hand, it is also possible that mature cells may transform into CSCs. The pancreas is composed of endocrine cells (*α*-cells, *β*-cells, etc.), acinar cells, and ductal cells, which all derive from a common progenitor expressing Pdx1 [43]. Both ductal cells and acinar cells have been proposed as cellular origins for the development of pancreatic cancer [44, 45]. Under certain conditions, pancreatic ductal cells or acinar cells acquire genetic alterations and dedifferentiate into pancreatic CSCs. Finally, pancreatic CSCs and their differentiated progeny contribute to tumor heterogeneity.

## 3. The Pancreatic CSC Niche

As is the case for normal stem cells, pancreatic CSCs require nutrients and signals from the surrounding microenvironment, also called pancreatic CSC niche, to achieve a dynamic balance between self-renewal and differentiation. As an anatomically distinct region within the TME, the pancreatic CSC niche is comprised of different types of cells and noncellular components, such as non-CSC cancer cells, cancer-associated fibroblasts (CAFs), pancreatic stellate cells (PSCs), immune cells, blood and lymphatic vessels, extracellular matrix (ECM), cytokines, chemokines, and growth factors [46].

Direct cell-cell interactions between pancreatic CSCs and stromal cells, as well as signaling pathways mediated through the expression and secretion of a range of growth factors and cytokines, play a key role in the regulation of pancreatic CSCs. PSCs can form a niche for CSCs to promote in vitro sphere formation and invasiveness by paracrine Nodal/Activin signaling [47]. TGF-*β* treatment significantly increases the proportion of pancreatic CSCs, which exhibit a high degree of epithelial-mesenchymal transition (EMT) and great invasion and migration activity in vitro [48]. Depletion of TAMs and inflammatory monocytes by inhibiting either the myeloid cell receptor colony-stimulating factor-1 receptor (CSF1R) or chemokine (C-C motif) receptor 2 (CCR2) decreases the number of pancreatic CSCs [49]. Another important contributor to the pancreatic CSC niche is CAFs. CAF-derived CXCL12 attracts CXCR4 expressing CSCs, and fibronectin secreted by fibroblasts promotes CSC attachment [50]. CAFs can stimulate stemness via activation of WNT and NOTCH pathways [51]. Pancreatic cancer is characterized by remarkable desmoplasia [52, 53]. CAF activation leads to the ECM remodelling [54, 55]. In normal tissues, the ECM has an effect on cell proliferation, differentiation, and migration [56]. Receptors expressed within the ECM allow stem cells to anchor to specific locations and communicate with surrounding cells within the niche. Loss of the ECM results in a decrease of stem cell numbers [57, 58]. The accumulation of the ECM in pancreatic cancer destroys the normal pancreatic architecture, promotes EMT, enhances CSC marker expression, and forms a barrier blocking therapeutics [59]. All these cellular and noncellular components establish a supportive niche to maintain the properties of CSCs and regulate their fate.

Targeting pancreatic cancer stroma is a promising new therapeutic option, but recent studies have spurred some controversy. Rhim et al. discovered that sonic hedgehog-deficient tumors had reduced fibroblast-rich desmoplastic stroma, aggressive behaviour, undifferentiated histology, increased vascularity, and heightened proliferation [60]. Ozdemir et al. found that depletion of CAFs and fibrosis led to enhanced numbers of pancreatic CSCs, immunosuppression, and reduced survival [61]. Saridegib is a small molecule targeting smoothened in the sonic hedgehog pathway. The inhibition of the hedgehog pathway depleted the tumor stroma, enhanced delivery of gemcitabine, and improved survival in a mouse model of pancreatic cancer [62]. However, a phase I/IIb trial of saridegib plus gemcitabine in patients with metastatic pancreatic cancer was stopped in 2012 because interim data showed that patients receiving the combination therapy had higher rates of progressive disease and lower overall survival than patients receiving placebo plus gemcitabine [63]. These findings suggest that some stromal elements might actually restrain tumor growth. Thus, the complex cross-talk between pancreatic cancer cells, including CSCs, and the stroma should be evaluated by further studies.

## 4. Resistance of Pancreatic CSCs to Chemotherapy

One key attribute of pancreatic CSCs is chemotherapy resistance, which may initially reduce the tumor bulk but fail to eradicate CSCs, resulting in recurrence of pancreatic cancer. Notably, resistance of pancreatic CSCs to chemotherapy is mediated by both intrinsic factors of CSCs and extrinsic factors of the CSC niche.

Cioffi et al. found that miR-17-92, targeting NODAL/ACTIVIN/TGF-*β*1/p21 signaling, was suppressed in gemcitabine-resistant pancreatic CSCs. Overexpression of miR-17-92 cluster or knockdown of p21 could inhibit chemoresistance of pancreatic CSCs [64]. The ATP-binding cassette (ABC) transporter, ABCG2, is an important source of drug resistance in cancer [65]. However, Bhagwandin et al. found that in pancreatic cancer, ABCG2 did not efflux gemcitabine and inhibition of ABCG2 did not sensitize pancreatic CSCs to gemcitabine [66]. Family with sequence similarity 83 member A (FAM83A) could promote pancreatic CSC-like traits by activating the Wnt/*β*-catenin and TGF-*β* signaling pathways and chemoresistance in pancreatic cancer. Inhibition of FAM83A significantly enhanced the sensitivity of pancreatic cancer to gemcitabine [67]. Our previous study also defined a distinguished group called side population (SP) cells from a metastatic human pancreatic cancer cell line with highly tumorigenic and metastatic characteristics after orthotopic injection. In particular, these SP cells showed properties of pancreatic CSCs. Wnt, NOTCH, and EGFR signaling pathways associated with CSCs were altered in SP cells. The proportion of SP cells was significantly enriched when cultured with increasing concentrations of gemcitabine [68]. In addition, as a part of the TME, the pancreatic CSC niche also contributes to chemoresistance. Extensive fibrosis produced by PSCs results in significant hypoxia in the pancreatic CSC niche. In turn, hypoxia stimulates PSCs to induce fibrosis and angiogenesis [69]. This impairs drug delivery and stimulates EMT, promoting chemoresistance of pancreatic cancer cells [70]. In addition, aberrant accumulation of ECM in the pancreatic CSC niche can reduce the penetration of chemotherapeutic agents [71].

## 5. The Potential Role of Pancreatic CSCs in Cancer Immunoediting

**Figure 2:**
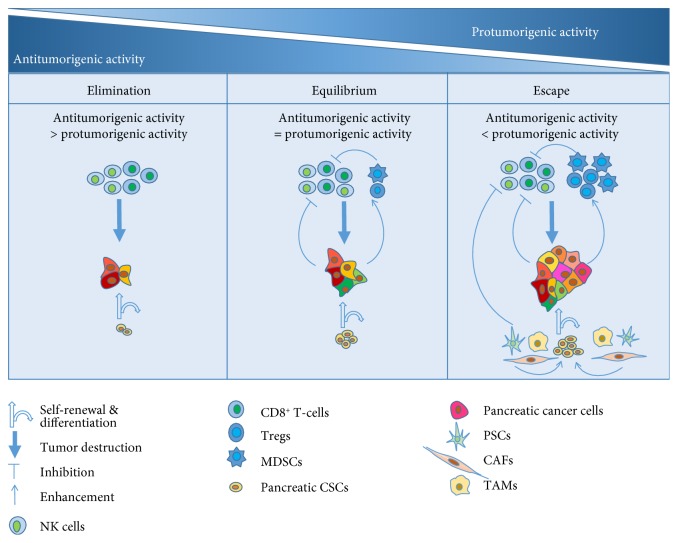
The potential role of pancreatic CSCs in cancer immunoediting. Elimination (left): in the elimination process, most of pancreatic cancer cells can be successfully detected and destroyed by the innate and adaptive system. However, pancreatic CSCs are believed to be immunologically privileged like normal stem cells. Low immunogenicity prevents pancreatic CSCs from recognition and elimination by the host immune system. Equilibrium (middle): in the equilibrium process, the immune system and pancreatic cancer cells that have survived the elimination process enter into a dynamic equilibrium. The function of the immune system can be negatively regulated by cancer cells and stromal cells. The majority of pancreatic cancer cells are destroyed, but some cancer cells acquire the ability to avoid immune destruction. The equilibrium process is functionally similar to the state of tumor dormancy. Escape (right): in the escape process, pancreatic cancer cells can inhibit host anticancer immunity by secretion of immunosuppressive factors and by recruitment of stromal cells, such as Tregs and MDSCs. Besides, PSCs, CAFs, and TAMs also support pancreatic CSC growth and promote immunosuppression. The immunosuppressive niche allows pancreatic CSCs to rapidly produce specialized cancer cells with high metastatic potential or chemoresistance. Finally, pancreatic CSCs and their differentiated progeny progressively grow into a visible tumor in the pancreas and even metastasize to distant sites.

Evading immune destruction is considered as a hallmark of cancer, but the mechanisms are not yet fully understood [72, 73]. The concept of cancer immunoediting describes the dynamic interaction between cancer and immune cells during cancer progression. Cancer immunoediting consists of three stages: elimination, equilibrium, and escape [74–76]. New mechanisms of immune escape are continuously discovered and translated to preclinical and clinical studies. Increasing studies have focused on the cross-talk between CSCs and immune cells, and recent findings raise the possibility that CSCs might get involved in the process of cancer immunoediting [75, 76]. Here, we speculate the potential role of pancreatic CSCs in different stages of cancer immunoediting (Figure 2).

In the elimination process, both innate and adaptive immune cells play a critical role in cancer immunosurveillance [77]. Several driver genes have been identified in pancreatic cancer, including tumor suppressor genes *CDKN2A*, *SMAD4*, and *TP53* and the oncogene *KRAS* [78–80]. Although immune response has been described to some of these antigens, the majority of T-cell antigens are located outside of classical driver mutations [81]. During pancreatic cancer initiation, malignant cells with these genetic mutations can upregulate activating NK cell receptor ligands and downregulate inhibitory ligands. For example, major histocompatibility complex class I-related chains A and B (MICA/B) are frequently expressed on the surface of pancreatic cancer cells. Such ligands bind to NKG2D on NK cells and other immune cells, activating NK cell cytotoxicity and leading to the release of proinflammatory cytokines, which facilitate the anticancer immune response [82]. Tumor-specific CD8^+^ T-cells can recognize and eliminate pancreatic cancer cells expressing tumor-associated antigens [83]. However, pancreatic CSCs exhibit a quiescent behaviour and low immunogenicity, which probably makes them the right candidate to escape immune surveillance [84, 85].

In the equilibrium process, immune response and pancreatic cancer progression are balanced [86]. The quiescent behaviour and longevity of pancreatic CSCs makes it easy to accumulate genetic and epigenetic alterations and survive the equilibrium process [87]. Upon asymmetric division, a cancer stem cell generates a daughter stem cell for self-renewal and a daughter cell that undergoes further differentiation. The differentiated pancreatic cancer cells are subjected to immunosurveillance, and most of them could be detected and destroyed by the immune system as mentioned above. In contrast, poorly immunogenic cancer cells are more likely to escape from immunosurveillance. In breast cancer, the downregulation of MICA/MICB on CSCs promotes the resistance of breast CSCs to NK cell cytotoxicity and lung metastasis formation [88]. Whether pancreatic CSCs survive by this mechanism needs to be explored. In the meanwhile, the pancreatic CSC niche is not totally established yet. The dependence of pancreatic CSCs on their niche may restrain their rapid propagation [89]. The equilibrium process is functionally similar to the state of tumor dormancy [90]. The pancreatic CSCs may stay dormant for a long time before eventually becoming clinically apparent.

In the escape process, pancreatic cancer cells successfully evade immune destruction. Several factors can result in the weakening of the immune system, such as aging, immunosuppressive drugs, and systemic immunosuppression. On the other hand, the TME of pancreatic cancer is generally regarded as poorly immunogenic and could also contribute to immune escape of pancreatic CSCs [91]. Pancreatic cancer cells are able to reprogram the TME via secretion of immunosuppressive factors and recruitment of immunosuppressive cells, such as regulatory T-cells (Tregs) and myeloid-derived suppressor cells (MDSCs), both of which can suppress the cytotoxicity of CD8^+^ T-cells and NK cells [92–94]. Monocytic MDSCs increase the frequency of ALDH1 (Bright) pancreatic CSCs and promote mesenchymal features of pancreatic cancer cells through tumor-induced STAT3 activation [95]. Besides, as mentioned above, PSCs, CAFs, and TAMs can also support pancreatic CSCs growth and promote immunosuppression in the niche. The immunosuppressive niche allows pancreatic CSCs to rapidly produce specialized cancer cells with high metastatic potential or chemoresistance. Finally, pancreatic CSCs and their differentiated progeny progressively grow into a visible tumor in the pancreas and even metastasize to distant sites.

Although the biological properties of pancreatic CSCs may help to explain how pancreatic cancer avoid immune destruction, the underlying mechanisms of pancreatic CSCs in cancer immunoediting remain to be further investigated.

## 6. Conclusion

Remarkable research results have been made in identifying characteristics of CSCs in pancreatic cancer over the last decade. Pancreatic CSCs have been suggested to exhibit high resistance to current therapies. However, there has been limited progress in developing alternative therapeutic options to eradicate pancreatic CSCs. Recently, cancer immunotherapy has emerged as an attractive research field in cancer treatment. Immune checkpoint inhibitors targeting CTLA-4, PD-1, and PD-L1 have shown clinical benefit in patients with advanced melanoma, non-small-cell lung cancer, and several other cancers [96–98]. Several phase I/II clinical trials studying the safety and efficacy of immune checkpoint inhibitors are being conducted in pancreatic cancer. In spite of efficacy in mismatch repair-deficient patients, the response is very poor [99, 100]. Due to the potential role of pancreatic CSCs in cancer immunoediting, immunotherapy targeting pancreatic CSCs and the niche components may provide a novel treatment strategy for pancreatic cancer [101, 102].

Pancreatic CSCs express specific markers, including CD24, CD44, CD133, EpCAM, CXCR4, c-Met, and CD166, at levels substantially different from the bulk pancreatic cancer cells. These markers not only have proven useful for identification and isolation of pancreatic CSCs but also can be considered as potential targets for cancer immunotherapy [103]. In addition, targeting the niche components may also help to eliminate CSCs [104]. Schatton et al. reported that CSCs inhibited T-cell activation by expression of PD-1 and B7.2 in melanoma [105, 106]. Lee et al. demonstrated preferential expression of PD-L1 on CSCs in head and neck cancer [107]. These findings raise the possibility that pancreatic CSCs might actively suppress anticancer immunity through CTLA-4 and PD-1 pathways. Assessment of the expression of immune checkpoint molecules on pancreatic CSCs and their niche will be necessary to verify whether this is the case in pancreatic cancer. In addition, Ames et al. found that NK cells preferentially killed pancreatic CSCs in vitro and intratumoral injection of activated NK cells in the human pancreatic cancer-bearing NSG mice significantly reduced the number of pancreatic CSCs and tumor burden [108].

Therefore, immunotherapy targeting pancreatic CSCs and their niche holds tremendous promise in pancreatic cancer treatment. Further research is urgently needed to improve our understanding of pancreatic CSCs and to develop more effective therapeutic strategies to eradicate pancreatic CSCs.
